# Physiological Characteristics of Incoming Freshmen Field Players in a Men’s Division I Collegiate Soccer Team

**DOI:** 10.3390/sports4020034

**Published:** 2016-06-08

**Authors:** Robert G. Lockie, DeShaun L. Davis, Samantha A. Birmingham-Babauta, Megan D. Beiley, Jillian M. Hurley, Alyssa A. Stage, John J. Stokes, Tricia M. Tomita, Ibett A. Torne, Adrina Lazar

**Affiliations:** Department of Kinesiology, California State University, Northridge, CA 91330, USA; deshaun.davis.907@my.csun.edu (D.L.D.); samantha.birminghambabauta.162@my.csun.edu (S.A.B.-B.); megan.beiley.78@my.csun.edu (M.D.B.); jillian.hurley.288@my.csun.edu (J.M.H.); alyssa.stage.634@my.csun.edu (A.A.S.); john.stokes.91@my.csun.edu (J.J.S.); tricia.tomita.228@my.csun.edu (T.M.T.); ibett.torne.734@my.csun.edu (I.A.T.); adrina.lazar.957@my.csun.edu (A.L.)

**Keywords:** association football, acceleration, maximal speed, high-intensity running, lower-body power, jump testing, college sports

## Abstract

Freshmen college soccer players will have lower training ages than their experienced teammates (sophomores, juniors, seniors). How this is reflected in field test performance is not known. Freshmen (*n* = 7) and experienced (*n* = 10) male field soccer players from the same Division I school completed soccer-specific tests to identify potential differences in incoming freshmen. Testing included: vertical jump (VJ), standing broad jump, and triple hop (TH); 30-m sprint, (0–5, 5–10, 0–10, and 0–30 m intervals); 505 change-of-direction test; Yo-Yo Intermittent Recovery Test Level 2 (YYIRT2); and 6 × 30-m sprints to measure repeated-sprint ability. A MANOVA with Bonferroni *post hoc* was conducted on the performance test data, and effect sizes and z-scores were calculated from the results for magnitude-based inference. There were no significant between-group differences in the performance tests. There were moderate effects for the differences in VJ height, left-leg TH, 0–5, 0–10 and 0–30 m sprint intervals, and YYIRT2 (*d* = 0.63–1.18), with experienced players being superior. According to z-score data, freshmen had meaningful differences below the squad mean in the 30-m sprint, YYIRT2, and jump tests. Freshmen soccer players may need to develop linear speed, high-intensity running, and jump performance upon entering a collegiate program.

## 1. Introduction

Participation rates in collegiate men’s soccer has increased noticeably from the 1980s and 1990s to the 2000s. In the academic year of 2011–2012, there were over 800 men’s teams that featured almost 23,000 players [1]. This volume of players ensures great competition amongst the schools that play collegiate soccer. In order to ensure long-term success for their team in this environment, collegiate coaches from all sports attempt to recruit the best athletes to their respective programs [2]. This will generally involve recruiting players from high school. Once a player is recruited into a soccer program, the coaching and strength and conditioning staff must physically prepare them for the rigors of the collegiate season. 

Players straight from high school competing within their first year at university may need more time to positively impact a soccer program. Young athletes will often need to physically mature [3], and will generally have lower training ages in most practice modalities (e.g., high-intensity running and sprinting, strength and power training) when compared to other players on their team [4]. Collegiate athletes are commonly defined by how many years they have been within a program. The general definitions are: freshmen (<2 years); sophomores (3 years); juniors (4 years); and seniors (5 years) [5]. Therefore, freshmen players may be further behind in their physical development when compared to their more experienced teammates. 

A further issue for collegiate soccer players is that they will only have a few weeks of pre-season training before they transition into the competitive season [6]. For soccer, this is particularly challenging as this sport places a great demand on a number of different physiological capacities. As a result, field testing batteries for soccer will generally incorporate a range of physiological assessments. This can include assessments of: lower-body power via jump tests, as jumping ability is important for challenging the ball when it is in the air [7], and leg power serves as a foundation for sprinting speed [8]; linear and change-of-direction (COD) speed, which are important skills for attaining ball possession and taking advantage of goal scoring chances [9]; and tests of maximal aerobic capacity and repeated-sprint ability (RSA), which relate to distances covered during a match [10]. These assessments would be useful for the coaching staff in order to understand the characteristics in freshmen soccer players, such that any weaknesses can be targeted during the abbreviated pre-season period.

Therefore, this study investigated the physiological characteristics of freshmen soccer players from a Division I men’s program, in order to compare them to experienced players (*i.e.*, sophomores, juniors, and seniors) from the same squad. Only field players were analyzed, due to the dissimilar movement patterns completed by goalkeepers [6,11]. Players were assessed in a bilateral vertical jump (VJ) to indirectly assess leg power in the vertical plane [7,12]; a bilateral and unilateral standing broad jump (SBJ) and triple-hop (TH) from each leg, to indirectly assess leg power in the horizontal plane [13,14]; a 30-m sprint (0–5 m, 0–10 m, and 0–30 m intervals), to assess acceleration and maximum velocity specific to soccer [15,16,17]; the 505 to measure COD ability [14,15,18]; the Yo-Yo Intermittent Recovery Test level 2 (YYIRT2) to assess soccer-specific aerobic endurance and high-intensity running performance [10,19]; and an RSA test that involved seven 30-m sprints completed on 20-s cycles [20,21]. The hypothesis was that freshmen would perform poorer in each of the field tests when compared to the experienced players. The results from this study will demonstrate those physiological capacities that could be targeted in freshmen soccer players to enhance their development in a Division I collegiate men’s team. 

## 2. Materials and Methods

### 2.1. Subjects

Seventeen field players (age: 20.41 ± 1.54 years; height = 1.81 ± 0.06 m; body mass = 77.54 ± 6.45 kg) from a Division 1 collegiate men’s soccer team were recruited for this study. The subjects were required to be: a member of the playing squad and a field player (*i.e.*, defender, midfielder, or forward); over 18 years of age; and injury-free and currently completing full training at the time of testing. As stated, goalkeepers were excluded from this analysis due to dissimilar movement patterns when compared to field players [6,11]. Players were defined by their level of experience and eligibility based on how long they had been in the soccer program, using procedures adapted from Chapman, Whitehead, and Binkert [5]. Two group classifications were used: freshmen (less than two years) and experienced (two years or greater). This resulted in seven players being in the freshmen group (age: 19.21 ± 1.11 years; height = 1.82 ± 0.05 m; body mass = 79.57 ± 8.72 kg), and 10 players in the experienced group (age: 21.20 ± 1.32 years; height = 1.80 ± 0.07 m; body mass = 76.11 ± 4.21 kg). G*Power software (v3.1.9.2, Universität Kiel, Germany) was used to confirm that the sample size of 17 was sufficient for a multivariate analysis of variance (MANOVA), between factors analysis, and ensured the data could be interpreted with a moderate effect level of 0.70 [22,23,24], and a power level of 0.80 when significance was set at 0.05 [25]. These parameters were set as standardized thresholds for the range of physical performance tests utilized in this research [23,24]. The data used in this study arose as a condition of player monitoring in which player activities were measured over the course of the pre-season [26]. As a result, the institutional ethics committee approved the use and analysis of pre-existing data. The study still conformed to the recommendations of the Declaration of Helsinki, and all subjects received a clear explanation of the study, including the risks and benefits of participation. Each player had also completed the university-mandated physical examination, and read and signed the university consent and medical forms for participation in collegiate athletics. 

### 2.2. Procedures

Testing was incorporated within the team’s gym and field sessions that occurred across three weeks in February. All subjects were familiar with the tests performed in this study, as they were consistently used by the team’s training staff for general player monitoring. The jump assessments were completed within one gym session, which took approximately 30–45 min to complete. The running assessments were completed prior to field training sessions following the team’s usual warm-up. Two field testing sessions were completed, which incorporated the: (1) 30-m sprint, 505 COD speed test, and RSA test; and (2) YYIRT2. The first field session lasted for approximately 60–75 min in duration, while the second field session took approximately 45–60 min. The testing was conducted to fit into the schedule designed by the team’s coaching staff, and 48–72 h was provided between each session. In the gym-based session for the jump testing, subjects wore their own athletic trainers, and testing was conducted on a rubber-matted floor. Field testing was conducted on a grass outdoor soccer pitch, and subjects wore their own cleats they used in competition.

Prior to data collection in the first gym testing session, the subject’s age, height, and body mass were recorded. Height was measured barefoot using a portable stadiometer (seca, Hamburg, Germany). Body mass was recorded by electronic digital scales (Tanita Corporation, Tokyo, Japan). The gym-based session was preceded by a standardized warm-up designed by the team’s coaching staff, consisting of 10 min of jogging, and 10 min of dynamic stretching of the lower limbs. Subjects also completed a standardized warm-up before each field session that was designed by the team’s coaching staff, which consisted of 10 min of jogging, 10 min of dynamic stretching of the lower limbs, and linear and lateral runs over 20–30 m that progressively increased in intensity. Examples of the dynamic stretches included walking quadriceps, hamstrings, gluteal, hip flexor, and groin stretches, as well as other dynamic movements, such as running technique drills. This dynamic warm-up followed procedures conducted in previous research that investigated maximal running tests [14,27], thus, the subjects were appropriately prepared for the assessment tasks that were to follow. Subjects completed testing in the order stated in this section, and rotated alphabetically by surname for each test [27], except for the YYIRT2, which was completed as a group. This ensured sufficient recovery periods (*i.e.*, greater than 3 min) between efforts in tests involving multiple trials. A standard metric tape measure was used to measure all distances.

### 2.3. Vertical Jump (VJ) 

The VJ was measured via a jump mat (Just Jump, Probotics Inc., Huntsville, AL, USA) [28]. The subject initially stood on the jump mat keeping their heels on the mat, before completing a countermovement and jumping as high as possible. No preparatory step was used, and no restrictions were placed on the lower-body range of motion during the countermovement. Subjects were free to swing their arms during the jump. This is more practical from a sports perspective, as the arms would generally always be used when jumping during a match to add angular momentum to the jump. Subjects were instructed to maintain straight legs during the flight, before landing on both feet with flexion of the hips, knees, and ankles. Within the software for the mat, jump height was calculated from flight time via the following equation: Jump Height = [½ × acceleration due to gravity (−9.81 m·s^2^) × (total flight time/2)^2^] [28]. Each subject completed two trials, and the best trial was used for analysis. The Lewis nomogram was used to calculate the power index [Power (kg·m·s^−1^) = $\sqrt{4.9}$ Body Mass $\sqrt{\text{vertical jump height}}$] [29]. A power index was utilized in this study to standardize for the influence of body mass on VJ height such that heavier subjects would not be penalized [29]. 

### 2.4. Standing Broad Jump (SBJ) 

The bilateral and unilateral SBJ was performed according to established methods [14]. For the bilateral SBJ, the subject placed the toes of both feet on the back of the starting line, and with a simultaneous arm swing and crouch, then jumped forward as far forward as possible, before finishing with a two-footed landing. Subjects had to execute a correct landing (*i.e.*, “stick” the landing) for the trial to be counted; if not, the trial was disregarded and reattempted. Generally, no more than 1–2 reattempts were required, and the majority of subjects did not need this. No restrictions were placed on the range of the countermovement, or the degree of arm swing used. The distance was measured using a standard tape measure, which was the perpendicular line from the front of the start line to the posterior surface of the back heel at the landing [13,14]. Following the bilateral jumps, subjects completed unilateral jumps in the same manner, for both the left and right legs [14]. Subjects took off from one leg, and then landed on both feet. The distance jumped was measured in the same manner as the bilateral SBJ, and the order of which leg was tested first was randomized. Each subject completed two trials for each jump condition, and the best trial was used. 

### 2.5. Triple Hop (TH)

The TH was used to assess unilateral lower-body functional power [13], and the methods were adapted from Hamilton, Shultz, Schmitz, and Perrin [13]. Similar to the unilateral SBJ, subjects started on one leg with their toes behind the start line. Subjects then completed three consecutive maximal hops, and were required to “stick” the landing on the final hop, and the other leg was not to have any contact with the ground. Failure to do so disqualified the trial, which was reattempted. No more than 1–2 reattempts were generally required for certain subjects. The distance was measured with a standard tape measure from the front of the start line to the posterior surface of the back heel of the leg at the landing [13,14]. Each leg was tested in an alternating order for two trials (four trials total), the order of which was randomized amongst the subjects. 

### 2.6. 30-m Sprint

30-m sprint time was recorded by a timing lights system (Fusion Sports, Coopers Plains, Australia). This timing lights were a single-beam system with microprocessor functionality and error correction processing [30], and previous research has indicated the reliability of sprint testing using this system [31]. Gates were positioned at 0 m, 5 m, 10 m, and 30 m, to measure the 0–5 m, 5–10 m, 0–10 m, and 0–30 m intervals. Sprints over 5 m [15], 10 m [16], and 30 m [17] have been used in the assessment of soccer players. The 0–5 m, 5–10 m, and 0–10 m intervals measured acceleration [8]; the 0–30 m time affords a measure of maximum velocity specific to soccer [17]. Gate height was set at 1.2 m, with a width of 2.5 m, and subjects began the sprint from a standing start 50 cm behind the start line to trigger the first gate. Subjects started at their own time once ready, and they were instructed to run maximally after they initiated their sprint. Subjects completed two trials [3,8], time for each interval was recorded to the nearest 0.001 s, and the fastest trial was used for analysis. 

### 2.7. 505 Change-of-Direction (COD) Speed Test

**Figure 1:**
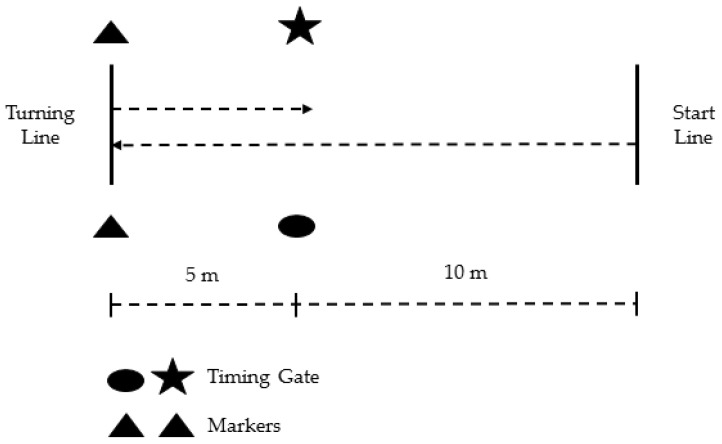
Layout of the 505 COD test. Subjects sprinted over the 10-m distance through the timing gate and over the 5-m distance to the turning line. They then planted either their left or right foot on the turning line to stop and change direction, before sprinting back through the timing gate.

The methodology for the 505 was used per established methods [14,15,18] with the set-up is shown in Figure 1. Subjects used a standing start with the same body position as the 30-m sprint. The subjects sprinted through the timing gate to the turning line, indicated by a line marked on the grass pitch. Subjects were to place either the left or right foot, depending on the trial, on or behind the turning line, before sprinting back through the gate. Two trials [3,8] were recorded for turns off the left and right side (four trials total), the order of which was randomized amongst the subjects. A researcher was positioned at the turning line, and if the subject changed direction before hitting the turning point, or turned off of the incorrect foot, the trial was disregarded and reattempted after the recovery period. Generally, only 1–2 reattempted trials for those subjects that did miss a trial. Time for each distance was recorded to the nearest 0.001 s, and the fastest trial for each leg was used for analysis. 

### 2.8. Yo-Yo Intermittent Recovery Test Level 2 (YYIIRT2)

The YYIRT2 was conducted according to established procedures [10,19]. The test consisted of repeated 2 × 20 m runs at a progressively increased speed. This was controlled by audio beeps from an iPad handheld device (Apple Inc., Cupertino, CA, USA) connected via Bluetooth to a potable speaker (QFX, Inc., Vernon, CA, USA) located immediately adjacent to 20-m long running lanes indicated by markers. Between each running bout, the participants had a 10-s rest period in which they were required to move to a cone 5 m away before returning to the start line. The YYIRT2 started at a speed of 13 kilometers per hour (km·h^−1^), which then increased by 2 km·h^−1^ after the first stage, and 1 km·h^−1^ after the second stage. The test then continued with stepwise 0.5 km·h^−1^ speed increments after every stage until exhaustion. The YYIRT2 was terminated for a subject when they failed to reach the finish line in time on two successive occasions or by volitional exhaustion. This was monitored by the coaching and support staff, and the performance value was recorded as the last completed running bout. All players performed the test within the session. Strong verbal encouragement was provided to players throughout the running of the test.

### 2.9. Repeated-Sprint Ability (RSA) Test

**Figure 2:**
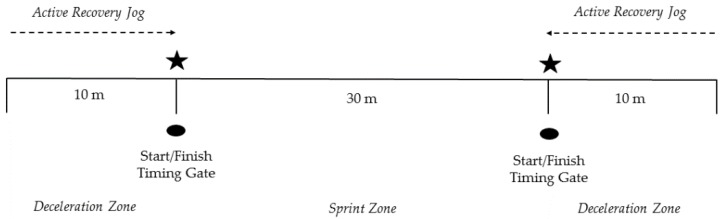
Layout of the RSA test.

The RSA test used in this research was adapted from the literature [20,21]. The test consisted of seven 30-m sprints completed on 20-s cycles, shown in Figure 1. Timing lights (Fusion Sports, Coopers Plains, Australia) were used to record the time for each sprint effort to the nearest 0.001 s, and were positioned at the start/finish positions at each end of the sprint zone. The coaches and support staff recorded the recovery intervals with a stopwatch (Accusplit, Pleasanton, CA, USA). After subjects passed through the second timing gate, they decelerated to a marker positioned 10 m past the finish line. This was followed by a 10-m active recovery jog back to the start/finish line for the next sprint (Figure 2). Subjects were required to be stationary at the start position 5 s prior to executing the next sprint, and were provided with feedback as to the progression of the recovery period. Strong verbal encouragement was provided to all subjects by the researchers. 

Two variables were calculated for the RSA test. They are:

- TT: the sum of all seven sprint times, expressed in s [20,21].
- PD: the decrement in sprint performance was measured as the percent change in sprint time from the first to last sprint [21]. This was calculated via the equation: Percent change (%) = [(last sprint time − first sprint time)/first sprint time] × 100.

### 2.10. Statistical Analysis

All statistical analyses were computed using the Statistics Package for Social Sciences (Version 22.0; IBM Corporation, New York, NY, USA), and stem-and-leaf plots were used to confirm a normal distribution in the data for each variable. As previously stated, players were placed in groups corresponding to their year of eligibility or experience; freshmen (<2 years’ experience) or experienced (≥2 years’ experience). Descriptive statistics (mean ± standard deviation [SD]; 95% confidence intervals) were calculated for each test parameter. A MANOVA, with Bonferroni *post hoc* for multiple comparisons, computed any significant differences between age, height, and body mass, the jump assessments, 30-m sprint, 505, YYIRT2, and RSA test measurements. Significance was set at *p* < 0.05. Effect sizes (*d*) were also calculated for the between-group comparison, where the difference between the means was divided by the pooled SD [32]. In accordance with Hopkins [22], a *d* less than 0.2 was considered a trivial effect; 0.2 to 0.6 a small effect; 0.6 to 1.2 a moderate effect; 1.2 to 2.0 a large effect; 2.0 to 4.0 a very large effect; and 4.0 and above an extremely large effect. Effect sizes were included in this study to ascertain how much difference existed between the groups irrespective of the *p* value [23,33], and to provide useful and practical information for the coach and practitioner [23,24].

As significant changes in aspects of athletic performance can be difficult to elicit in trained athletes with longitudinal training [34], this study also incorporated an analysis of worthwhile differences. Buchheit [23] has noted that significance does not inform on magnitude of effects, despite the importance of magnitude. Therefore, this magnitude-based inference analysis was included. The test data for each subject were converted to z-scores, via the formula: z-score = (athlete’s test score − average score from the squad)/SD [35]. In accordance with previous recommendations [35] and research [18], the squad mean (*n* = 17) was used in the calculation of the z-score. When appropriate, absolute values for z-scores were derived (e.g., the typical sprint time z-score, where a faster performance equates to a lower sprint time, leads to a negative z-score, so this was converted into an absolute value), such that a positive score above zero represented a superior performance compared to the mean for all tests; a negative score was considered worse than the mean. The mean ± SD for the z-scores were calculated for the freshmen and experienced groups. A worthwhile difference in the z-score was either above or below the squad mean. This was determined by computing the smallest worthwhile change (SWC) in z-score for the test. The SWC for each test was calculated by multiplying the between-subject z-score SD by 0.2, which is the typical small effect [22]. As these were standardized scores, the SD is 1.0, and therefore the SWC is equals 0.2. Thus, those subjects that had a z-score difference (≥0.2 or −0.2) that exceeded the SWC either positively or negatively were deemed to have a meaningful difference in the particular test. These data are important because it also provides useful and practical information for the coach [23,24]. As for the performance test results, a MANOVA with Bonferroni *post hoc* calculated any between-group differences in the z-scores (*p* < 0.05), and effect sizes were calculated. Depending on the results from the z-score data, certain tests were investigated further via individual subject analysis.

## 3. Results 

Table 1 shows the physical characteristics for the freshmen and experienced players. The experienced group were significantly older than the freshmen, although there were no differences in height or body mass.

Table 2 displays performance test data. There were no significant differences in any of the jump tests, although there were moderate effects for the greater VJ and left-leg TH for the experienced group. No significant between-group differences were found for the 30-m sprint. There were moderate effects for the 0–5 m, 0–10 m and 0–30 m times, and a trivial effect for the 5–10 m interval. There were also no significant between-group differences in the 505 performed from each leg, nor RSA TT or PD. The greater YYIRT2 distance achieved by the experienced group had a moderate effect, although the difference was not significant.

When considering the z-score analysis for the performance tests (Table 3), there were, again, no significant differences recorded between the experienced and freshmen groups. However, there were numerous examples where a group had a worthwhile difference (z-score ≥ 0.2 or −0.2) above or below the squad mean. The freshmen had a worthwhile difference below the squad mean in each of the jump tests, except VJ power index, the 0–5 m, 0–10 m, and 0–30 m sprint intervals, and the YYIRT2 distance. The experienced group had worthwhile performance differences above the squad mean in the VJ, SBJ, left- and right-leg TH, 0–5 m, 0–10 m, and 0–30 m sprint intervals, and the YYIRT2. The experienced group also had a worthwhile greater PD in the RSA test. Moderate (but non-significant) effects for between-group differences in z-scores were observed for the left-leg TH, the 0–5 m, 0–10 m, and 0–30 m sprint intervals, YYIRT2, and RSA PD. These variables were analyzed further via individual player z-scores.

**Figure 3:**
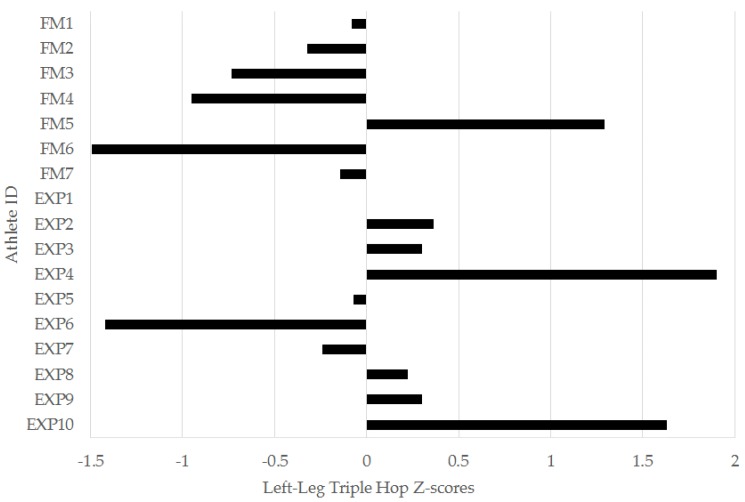
Individual left-leg triple hop z-scores for freshmen (FM) and experienced (EXP) players from a Division I collegiate men’s soccer team.

**Figure 4:**
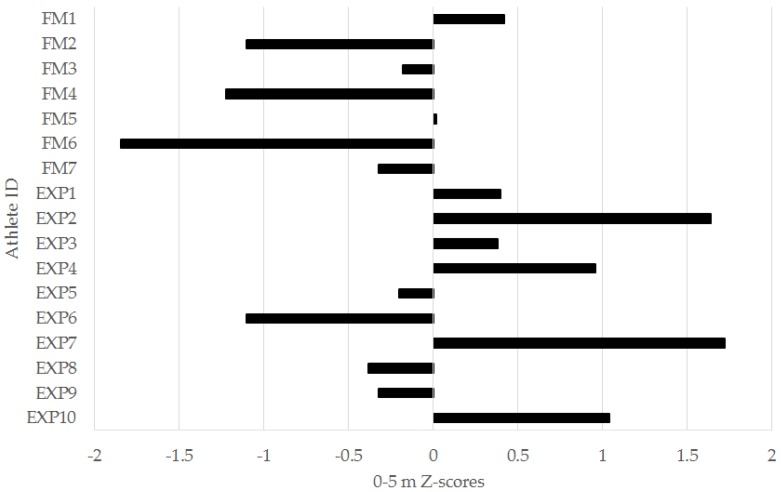
Individual 0–5 meter (m) sprint z-scores for freshmen (FM) and experienced (EXP) players from a Division I collegiate men’s soccer team.

**Figure 5:**
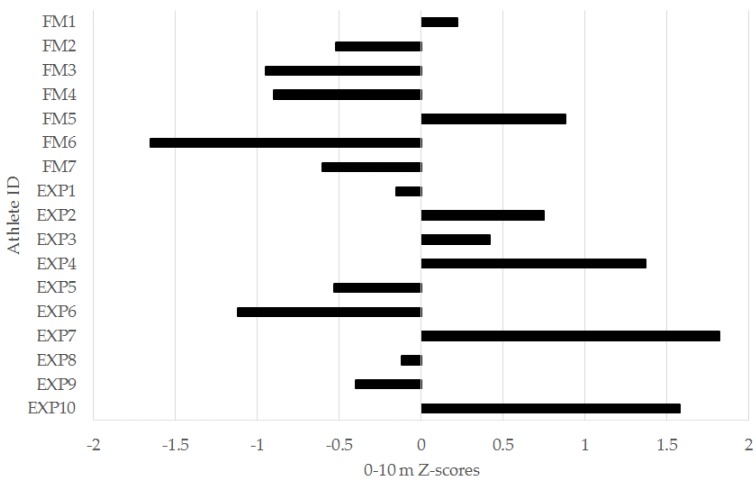
Individual 0–10 meter (m) sprint z-scores for freshmen (FM) and experienced (EXP) players from a Division I collegiate men’s soccer team.

In the left-leg TH, 6/7 (86%) freshmen were below the squad mean, while 6/10 (60%) of experienced players were above. For the 0–5 m (Figure 3), 0–10 m (Figure 4), and 0–30 m (Figure 5) sprint intervals, 5/7 (71%) of freshmen were below the squad mean. In the experienced players, 6/10 were superior to the mean in the 0–5 m and 0–30 m sprint intervals, while 5/10 (50%) were superior in the 0–10 m interval. In the YYIRT2, 6/7 freshmen performed poorer than the squad mean. 5/10 (50%) experienced players were superior to the mean. Only 2/7 (29%) freshmen were lower than the squad mean in RSA PD, while 7/10 (70%) experienced players were lower.

## 4. Discussion

This is the first study to analyze the differences between freshmen and experienced (*i.e.*, sophomores, juniors, and seniors) soccer field players from a Division I men’s team in performance tests specific to soccer. The results indicated there were few significant differences between the two groups, which is counter to the study’s hypothesis. Experienced players were older (although not taller or heavier; Table 1), and there were no significant differences in the performance tests. Magnitude-based inference analyses indicated some differences in the jump tests, 30-m sprint, YYIRT2, and RSA PD. The z-score analysis also suggested some meaningful differences below the squad mean for the freshmen players in the jump tests (VJ, SBJ, and TH), 30-m sprint, and YYIRT2. In addition to this, the z-score analysis also revealed that, although there was a greater percentage of freshmen players below the squad mean in the 30-m sprint and YYIRT2, there were also experienced players below the standard. The results from this study have implications for collegiate soccer and strength and conditioning coaches.

**Figure 6:**
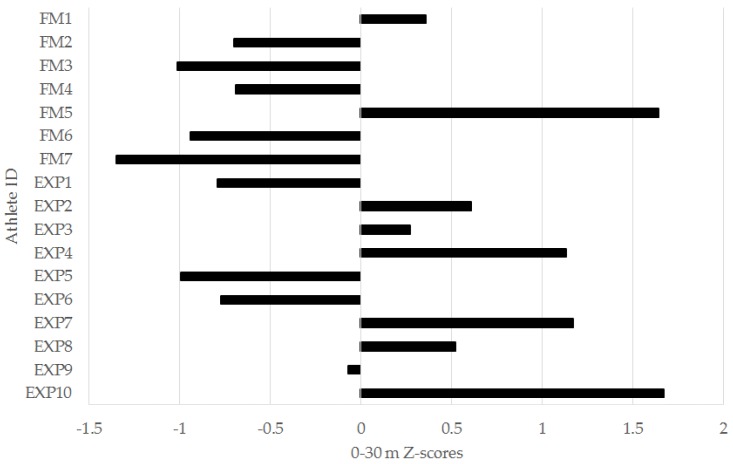
Individual 0–30 meter (m) sprint z-scores for freshmen (FM) and experienced (EXP) players from a Division I collegiate men’s soccer team.

There were no significant between-group differences in any of the 30-m sprint intervals (Table 1 and Table 2). However, it is important to consider magnitudes of difference in sport science research, as this provides the most practical information for a coach [23]. There were moderate effects for the faster 0–5 m, 0–10 m, and 0–30 m sprint intervals for the experienced group (Table 1 and Table 2). Additionally, 71% of the freshmen were below the squad mean in the 0–5 m (Figure 4), 0–10 m (Figure 5), and 0–30 m (Figure 6) sprint intervals. Linear speed is a key characteristic for soccer players, as faster running speeds during a match will allow players to position themselves for involvement in play and goal-scoring chances [9]. The results from this study suggest that experienced players from the analyzed squad may have a practically useful difference in linear speed when compared to freshmen [23,24,33]. Nevertheless, what should also be acknowledged is that when considering the individual player data for the 30-m sprint (Figure 4, Figure 5 and Figure 6), there were still experienced players who were below the squad mean in the different sprint intervals. Although improvements to linear speed could be a focus for incoming freshmen to a men’s collegiate soccer team, coaches should carefully monitor the sprinting capacity of experienced players as well.

**Figure 7:**
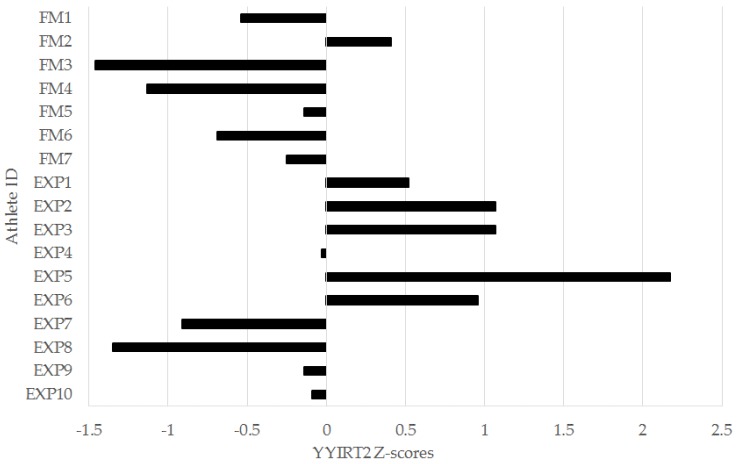
Individual Yo-Yo Intermittent Recovery Test Level 2 (YYIRT2) sprint z-scores for freshmen (FM) and experienced (EXP) players from a Division I collegiate men’s soccer team.

The YYIRT2 places great stress on both the aerobic and anaerobic capacity of an athlete, which is shown through reduced levels of creatine phosphate, and high muscle and blood lactate at the end of the test [10]. This provides evidence of the intensity of this assessment, and the YYIRT2 has been validated as a measure of high-intensity running performance in soccer players [19]. In this study, although not significant, there were moderate effects for the greater YYIRT2 distance covered by experienced players when compared to the freshmen group (Table 2 and Table 3; Figure 7). The magnitude of this difference [23,24,33] suggests that the experienced collegiate players in this study may have a better ability to tolerate the intensity of the YYIRT2 than the freshmen. Given that international-level soccer players perform better in the YYIRT2 when compared to their lesser counterparts [10], superior performance in the YYIRT2 should also benefit players at the college level. When considering the structure of the test, this could relate to a greater distance covered and more high-intensity running efforts performed during a match [10,19]. These results suggest that high-intensity running training could also be a focus for incoming freshmen to a collegiate men’s soccer program. Nevertheless, the individual z-scores for the YYIRT2 illustrated that there were still experienced players meaningfully below the mean for the squad (Figure 7). Strength and conditioning coaches should also ensure that all experienced players are up to standard in high-intensity running performance as measured by the YYIRT2. 

Lower-body power, which can be extrapolated from jump testing [8], is also an essential quality for soccer players. An effective VJ is needed when challenging for possession if the ball is in the air [36], while horizontal power, which can be inferred from SBJ and TH performance [13,14], has been linked to faster sprinting speeds in team sport athletes [27]. There were no significant differences between the two groups in any of the jump tests, and VJ power was almost identical between the two groups (Table 2). Nonetheless, magnitude-based inference [23,24,33] indicated meaningful differences below the squad mean in VJ height and the horizontal jump distances in freshmen players, and a moderate difference between the groups in the left-leg TH (Table 3). Indeed, 86% of freshmen performed worse than the squad mean in the left-leg TH (Figure 3). Hamilton, Shultz, Schmitz, and Perrin [13] found that TH distance positively related to both VJ height and isokinetic knee extensor strength in collegiate men’s and women’s soccer players, providing evidence of its validity as a lower-body performance test. Collectively, the z-score data suggests freshmen soccer players may need to improve jump performance and lower-body power upon their entry into a collegiate program, and this may be more evident in a unilateral jump test such as the TH.

There were no significant differences between the freshmen and experienced groups for 505 time from both legs (Table 2), and the z-scores were also close to the squad mean for both groups (Table 3). Within an elite English soccer game, Bloomfield, Polman, and O’Donoghue [11] reported that soccer players perform on average over 700 direction changes. It could be expected that a high volume of COD maneuvers would also occur within soccer matches at lower levels of play, such as high school and collegiate competition. As a result, both the freshmen and experienced players may be effectively conditioned in COD ability from prior training and soccer-playing experience, which could have influenced the study results. Further to this, the 505 only features one, 180° cut [14,15,18]. The number and angle of direction changes can affect how COD ability is assessed [31]. More complex COD tests may present different results in comparisons between freshmen and experienced collegiate men’s soccer players, which should be investigated in future research. 

**Figure 8:**
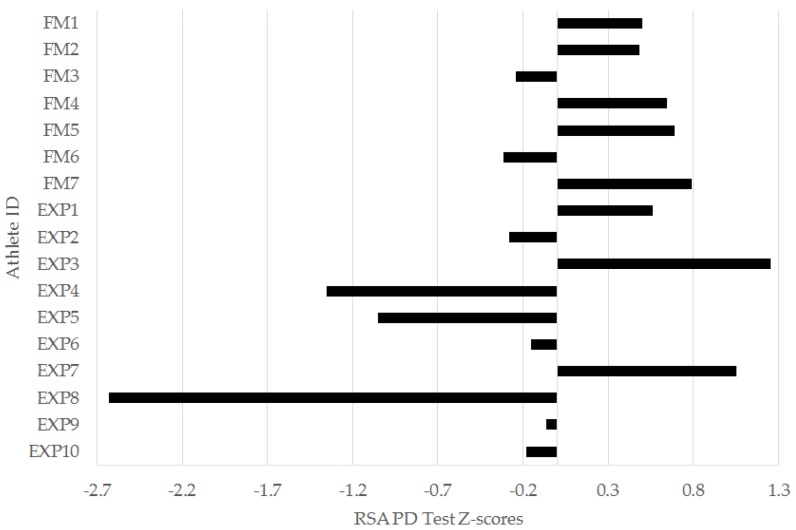
Individual sprint performance decrement from the repeated-sprint ability test (RSA PD) z-scores for freshmen (FM) and experienced (EXP) players from a Division I collegiate men’s soccer team.

Given the results from the 30-m sprint, it could be expected that there would also be differences in the RSA TT when comparing the freshmen and experienced groups. This is because a higher maximal sprinting velocity has been related to lower total times in RSA tests conducted on elite Norwegian [37] and Brazilian [38] male soccer players; however, this was not the case in this research (Table 2 and Table 3). The experienced players did have a worthwhile difference above the squad mean for RSA PD (Table 3 and Figure 8), which suggested a greater percentage time drop-off from the first to last sprint. Indeed, one experienced player had a large RSA PD z-score of −2.63. These data could potentially relate to a higher sprinting speed (and, thus, lower sprint time) achieved in the first sprint, which is understandable given the moderately [23,24,33] faster 30-m sprint time achieved by experienced players in the linear speed test (Table 2). Nonetheless, the difference in RSA PD was not significantly different to that from the freshmen group (Table 2 and Table 3). The results from this study suggest that the RSA test conducted did not differentiate between freshmen and experienced collegiate men’s soccer players from this study.

There are certain limitations with this research that should be discussed. Only one squad was analyzed (*n* = 17), and the sample size dictated using two groups in this study (*i.e.*, freshmen and experienced). A larger sample could have potentially allowed the analysis of players by each year of experience group (*i.e.*, freshmen, sophomores, juniors and seniors). Although analyzing the data via two groups increased the statistical power [25], future research should attempt to increase sample size to investigate any physiological and performance differences between male collegiate soccer players from each year of experience. No measures of lower-body strength were included in this study. The coaching staff for the soccer squad restricted the use of maximum strength testing, due to a perceived risk of injury during testing [39]. As lower-body strength has been related to superior jumping [12,40] and sprinting [40] performance in athletic populations, future research should measure the maximal strength of freshmen and experienced men’s collegiate soccer players. Only one form of COD speed (*i.e.*, the 505) and RSA (*i.e.*, 6 × 30-m sprints completed on 20-s cycles) test were measured in this research. There could potentially be a different response by freshmen and collegiate soccer players if other COD speed or RSA tests were used, and this could be elucidated in future studies. Nonetheless, within the context of these limitations, this study demonstrated that there are certain differences (*i.e.*, linear speed measured by a 30-m sprint and high-intensity running measured by the YYIRT2) between freshmen and experienced collegiate soccer players from a Division I men’s team.

## 5. Conclusions 

There were no significant differences in soccer-specific performance tests between freshman and experienced collegiate male players. However, magnitude-based inferences indicated some practical disparities in selected assessments that could have implications for freshmen players and their coaches. Linear speed could be targeted for improvement in freshmen soccer players. Additionally, high-intensity running performance, as measured by the YYIRT2, may need to be enhanced early in a freshmen collegiate players’ career. There would also likely be experienced players within a squad that need to improve their maximal sprinting and high-intensity running performance, and coaches should be cognizant of this. The study results also suggested that there were meaningfully lower performances in VJ height, and SBJ and TH distance, in addition to the 30-m sprint and YYIRT2, in the freshmen group. Thus, sprinting speed, high-intensity running, and jump performance could be a focus for freshmen male soccer players entering a collegiate program. 

## Figures and Tables

**Table 1:** Characteristics (mean ± SD; 95% confidence intervals) of freshmen and experienced players from a Division I collegiate men’s soccer team as measured by age, height, and body mass. m: meters; kg: kilograms.

Characteristics	Freshmen (*n* = 7)	Experienced (*n* = 10)	*p* Value	*d*	*d* Strength
Age (years)	19.29 ± 1.1	21.20 ± 1.32 *	0.046	1.57	Large
	(18.26–20.31)	(20.26–22.14)			
Height (m)	1.82 ± 0.0	1.80 ± 0.07	0.610	0.33	Small
	(1.77–1.86)	(1.76–1.85)			
Body Mass (kg)	79.57 ± 8.72	76.11 ± 4.21	0.367	0.51	Small
	(71.51–87.64)	(73.10–79.13)			

* Significantly different from the freshmen group (*p* < 0.05).

**Table 2:** Physiological characteristics (mean ± SD; 95% confidence intervals) of freshmen and experienced players from a Division I collegiate men’s soccer team as measured by: vertical jump (VJ); VJ power index (VJ PI); bilateral and unilateral (left and right legs) standing broad jump (SBJ); left- and right-leg triple hop (TH); 30-meter (m) sprint (0–5 m, 0–10 m, and 0–30 m intervals); 505 change-of-direction speed test from the left and right legs; Yo-Yo Intermittent Recovery Test Level 2 (YYIRT2); and total time (TT) and performance decrement from the first to last sprint (PD) from a repeated-sprint ability (RSA) test. kg·m·s^−1^ = kilogram meters per second; s = seconds; %: percentage.

Variables	Freshmen (*n* = 7)	Experienced (*n* = 10)	*p* Value	*d*	*d* Strength
VJ (m)	0.62 ± 0.08	0.67 ± 0.08	0.250	0.63	Moderate
	(0.55 to 0.70)	(0.61 to 0.72)			
VJ PI (kgm·s^−1^)	138.36 ± 13.30	137.38 ± 13.72	0.952	0.07	Trivial
	(126.06 to 150.67)	(127.56 to 147.19)			
SBJ (m)	2.33 ± 0.26	2.45 ± 0.21	0.324	0.51	Small
	(2.09 to 2.57)	(2.30 to 2.60)			
SBJ Left (m)	2.10 ± 0.16	2.18 ± 0.21	0.155	0.43	Small
	(1.95 to 2.24)	(1.98 to 2.30)			
SBJ Right (m)	2.07 ± 0.16	2.14 ± 0.22	0.082	0.36	Small
	(1.91 to 2.22)	(1.98–2.30)			
TH Left (m)	6.66 ± 0.52	7.04 ± 0.62	0.282	0.66	Moderate
	(6.18 to 7.13)	(6.51 to 7.56)			
TH Right (m)	6.80 ± 0.72	7.13 ± 0.78	0.244	0.44	Small
	(6.13 to 7.47)	(6.48 to 7.79)			
0–5 m (s)	1.030 ± 0.040	0.979 ± 0.046	0.060	1.18	Moderate
	(0.993 to 1.067)	(0.946 to 1.012)			
5–10 m (s)	0.712 ± 0.032	0.711 ± 0.027	0.524	0.03	Trivial
	(0.682 to 0.741)	(0.692 to 0.730)			
0–10 m (s)	1.742 ± 0.050	1.690 ± 0.059	0.097	0.95	Moderate
	(1.696 to 1.788)	(1.648 to 1.733)			
0–30 m (s)	4.150 ± 0.149	4.056 ± 0.131	0.157	0.67	Moderate
	(4.012 to 4.288)	(3.962 to 4.150)			
505 Left (s)	2.245 ± 0.068	2.235 ± 0.198	0.672	0.07	Trivial
	(2.182 to 2.308)	(2.083 to 2.387)			
505 Right (s)	2.186 ± 0.056	2.210 ± 0.105	0.340	0.29	Small
	(2.134 to 2.237)	(2.129 to 2.291)			
YYIRT2 (m)	893.33 ± 250.01	1210.00 ± 379.39	0.286	0.99	Moderate
	(630.96 to 1155.71)	(938.60 to 1481.40)			
RSA TT (s)	32.076 ± 1.310	31.674 ± 0.759	0.279	0.38	Small
	(30.865 to 33.288)	(31.091 to 32.257)			
RSA PD (%)	3.83 ± 1.93	6.61 ± 5.29	0.083	0.70	Moderate
	(2.04 to 5.61)	(2.55 to 10.67)			

**Table 3:** Z-scores (mean ± SD; 95% confidence intervals) and smallest worthwhile change (SWC) above or below the squad mean for freshmen and experienced players from a Division I collegiate men’s soccer team as measured by: vertical jump (VJ); bilateral and unilateral (left and right legs) standing broad jump (SBJ); left- and right-leg triple hop (TH); 30-meter (m) sprint (0–5 m, 0–10 m, and 0–30 m intervals); 505 change-of-direction speed test from the left and right legs; Yo-Yo Intermittent Recovery Test Level 2 (YYIRT2); and total time (TT) and performance decrement from the first to last sprint (PD) from a repeated-sprint ability (RSA) test.

Variables	Freshmen (*n* = 7)	Experienced (*n* = 10)	*p* Value	*d*	*d* Strength
VJ	−0.32 ± 0.96 §	0.21 ± 1.01 §	0.249	0.54	Small
	(−1.21 to 0.56)	(−0.51 to 0.94)			
VJ PI	0.04 ± 1.01	−0.03 ± 1.04	0.950	0.07	Trivial
	(−0.89 to 0.98)	(−0.78 to 0.71)			
SBJ	−0.28 ± 1.13 §	0.22 ± 0.91 §	0.324	0.49	Small
	(−1.33 to 0.76)	(−0.43 to 0.87)			
SBJ Left	−0.29 ± 0.85 §	0.17 ± 1.12	0.155	0.46	Small
	(−1.07 to 0.50)	(−0.63 to 0.98)			
SBJ Right	−0.22 ± 0.82 §	0.14 ± 1.12	0.082	0.37	Small
	(−0.98 to 0.54)	(−0.66 to 0.94)			
TH Left	−0.35 ± 0.85 §	0.30 ± 1.06 §	0.282	0.68	Moderate
	(−1.16 to 0.47)	(−0.59 to 1.18)			
TH Right	−0.24 ± 0.96 §	0.21 ± 1.04 §	0.243	0.45	Small
	(−1.13 to 0.65)	(−0.67 to 1.08)			
0–5 m	−0.60 ± 0.80 §	0.41 ± 0.93 §	0.060	1.16	Moderate
	(−1.34 to 0.14)	(−0.25 to 1.08)			
5–10 m	−0.04 ± 1.14	0.00 ± 0.97	0.520	0.04	Trivial
	(−1.09 to 1.02)	(−0.69 to 0.69)			
0–10 m	−0.50 ± 0.83 §	0.36 ± 0.99 §	0.096	0.94	Moderate
	(−1.27 to 0.26)	(−0.35 to 1.07)			
0–30 m	−0.38 ± 1.04 §	0.28 ± 0.92 §	0.157	0.67	Moderate
	(−1.35 to 0.58)	(−0.38 to 0.93)			
505 Left	−0.04 ± 0.45	0.03 ± 1.31	0.671	0.07	Trivial
	(−0.45 to 0.37)	(−0.98 to 1.04)			
505 Right	0.17 ± 0.66	−0.12 ± 1.24	0.342	0.29	Small
	(−0.44 to 0.78)	(−1.07 to 0.83)			
YYIRT2	−0.54 ± 0.69 §	0.33 ± 1.04 §	0.286	0.99	Moderate
	(−1.26 to 0.18)	(−0.42 to 1.07)			
RSA TT	−0.22 ± 1.29 §	0.17 ± 0.75	0.280	0.37	Small
	(−1.41 to 0.97)	(−0.40 to 0.75)			
RSA PD	0.36 ± 0.45 §	−0.28 ± 1.23 §	0.083	0.69	Moderate
	(−0.05 to 0.78)	(−1.23 to 0.66)			

§ SWC greater than 0.2 (superior to squad mean) or less than −0.2 (lower than squad mean).

## Abbreviations

The following abbreviations are used in this manuscript:

COD	Change-of-direction
RSA	Repeated-sprint ability
VJ	Vertical jump
SBJ	Standing broad jump
TH	Triple hop
m	Meter
YYIRT2	Yo-Yo Intermittent Recovery Test level 2
s	Second
km·h^−1^	Kilometers per hour
RSA TT	sum of all sprint times from the RSA test
PSA PD	sprint time decrement measured as percentage from first to last sprint in RSA test
SD	Standard deviation
*p*	Significance
*d*	Effect size
SWC	Smallest worthwhile change
FM	Freshmen
EXP	Experienced
